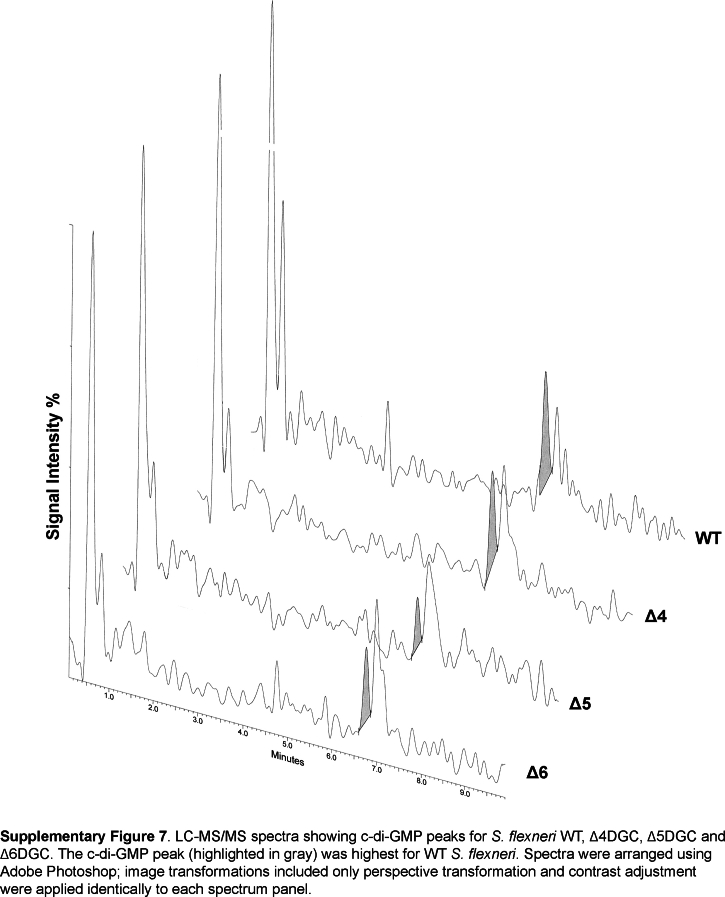# Intact and mutated *Shigella* diguanylate cyclases increase c-di-GMP

**DOI:** 10.1016/j.jbc.2024.107525

**Published:** 2024-07-01

**Authors:** Ruchi Ojha, Stefanie Krug, Prentiss Jones, Benjamin J. Koestler

**Affiliations:** 1 Department of Biological Sciences, Western Michigan University, Kalamazoo, Michigan, USA; 2 Department of Microbiology, University of Washington, Seattle, Washington, USA; 3 Department of Pathology, Western Michigan University Homer Stryker, M.D. School of Medicine, Kalamazoo, Michigan, USA

**Keywords:** cyclic di-GMP, diguanylate cyclase, Shigella, Escherichia, infection

## Abstract

The intracellular human pathogen *Shigella* invades the colonic epithelium to cause disease. Prior to invasion, this bacterium navigates through different environments within the human body, including the stomach and the small intestine. To adapt to changing environments, *Shigella* uses the bacterial second messenger cyclic di-GMP (c di-GMP) signaling system, synthesized by diguanylate cyclases (DGCs) encoding GGDEF domains. *Shigella flexneri* encodes a total of 9 GGDEF or GGDEF-EAL domain enzymes in its genome, but five of these genes have acquired mutations that presumably inactivated the c-di-GMP synthesis activity of these enzymes. In this study, we examined individual *S*. *flexneri* DGCs for their role in c-di-GMP synthesis and pathogenesis. We individually expressed each of the four intact DGCs in a *S. flexneri* strain, where these four DGCs had been deleted (Δ4DGC). We found that the 4 *S. flexneri* intact DGCs synthesize c-di-GMP at different levels *in vitro* and during infection of tissue-cultured cells. We also found that *dgcF* and *dgcI* expression significantly reduces invasion and plaque formation, and *dgcF* expression increases acid sensitivity, and that these phenotypes did not correspond with measured c-di-GMP levels. However, deletion of these four DGCs did not eliminate *S. flexneri* c-di-GMP, and we found that *dgcE*, *dgcQ*, and *dgcN*, which all have nonsense mutations prior to the GGDEF domain, still produce c-di-GMP. These *S. flexneri* degenerate DGC pseudogenes are expressed as multiple proteins, consistent with multiple start codons within the gene. We propose that both intact and degenerate DGCs contribute to *S. flexneri* c-di-GMP signaling.

Shigellosis, caused by the human pathogen *Shigella*, is a prominent gastrointestinal infection in developing countries (1, 2). *Shigella flexneri* evolved from commensal *Escherichia coli* to efficiently infect the human gastrointestinal tract after navigating different host microenvironments (3, 4). With the global rise in antibiotic resistance among *Shigella* strains (5, 6), and the lack of vaccines for Shigellosis prevention (1, 7), it is imperative to understand *Shigella* pathogenesis mechanisms. In the colon, *Shigella* invades the epithelium using a type III secretion system encoded on a ∼220kb virulence plasmid (7, 8, 9, 10). Once inside colonic epithelial cells, *Shigella* multiplies and spreads using actin-based motility (11, 12, 13).

During infection, *S. flexneri* senses and responds to many different environmental signals to adapt to various microenvironments within the human body. For example, lower pH in the stomach alters the expression of acid-related genes and induces biofilm formation (14, 15), bile acids in the small intestine promote initial adhesion and invasion (16, 17, 18), and formate within a host cell promotes cell-to-cell spread (19). Bacteria like *S. flexneri* encode many different systems to sense and respond to these signals, one of which is the second messenger cyclic di-GMP (c di-GMP) signaling system (20, 21).

C-di-GMP signaling is widely conserved in most bacteria (22, 23) and regulates diverse phenotypes (24, 25, 26, 27, 28). C-di-GMP synthesis is driven by diguanylate cyclases (DGCs) encoding a C-terminal GGDEF domain, which brings together two GTP molecules. Alterations of the GGDEF catalytic site can eliminate c-di-GMP synthesis of these enzymes (29, 30, 31). Conversely, c-di-GMP is degraded by c-di-GMP–specific phosphodiesterases encoding an EAL domain (22, 23, 28). In many bacteria, c-di-GMP promotes biofilm formation and reduces invasive capacity (7, 20, 32). However, certain DGCs and phosphodiesterases synthesize local pools of c-di-GMP, allowing them to regulate specific phenotypes regardless of overall c-di-GMP levels; this phenomenon is known as signaling specificity (31, 33, 34). *E. coli* K12 encodes 19 GGDEF-containing genes in its genome (35); in comparison, *S. flexneri* encodes nine GGDEF or GGDEF-EAL–containing genes, but the majority of these genes contain mutations that presumably eliminate c-di-GMP synthesis activity (pseudogenes) (36). *S. flexneri* has four DGCs predicted to synthesize c-di-GMP: *dgcC*, *dgcF*, *dgcI*, and *dgcP* (36).

We previously showed that deletion of intact *S. flexneri* DGCs alters pathogenesis-related phenotypes (20). Here, we investigate how *S. flexneri* DGCs contribute to c-di-GMP levels and how the expression of these genes impacts different phenotypes. To this end, we created a mutant *S. flexneri* strain where all four intact DGCs were deleted (Δ4DGC) and then expressed each *S. flexneri* DGC and respective active site mutant DGCs (GG→AA) from plasmids. We observed that all four DGCs were able to synthesize c-di-GMP at different levels and that each gene differentially contributed to acid resistance (AR), invasion, and plaque formation. Interestingly, we still detected c-di-GMP in our *S. flexneri* Δ4DGC strain, which led us to examine the five putative *S. flexneri* DGC pseudogenes. We found that expression of *S. flexneri dgcE, dgcQ,* and *dgcN*, each of which have nonsense mutations prior to the GGDEF domain, increased c-di-GMP, and deletion of *S. flexneri* Δ*dgcE* (Δ5DGC) and Δ*dgcE*Δ*dgcQ* (Δ6DGC) from the Δ4DGC strain significantly reduced c-di-GMP. This provides evidence that *S. flexneri* pseudogenes can retain function.

## Results

### *S. flexneri* DGCs increase c-di-GMP

**Figure S1:**
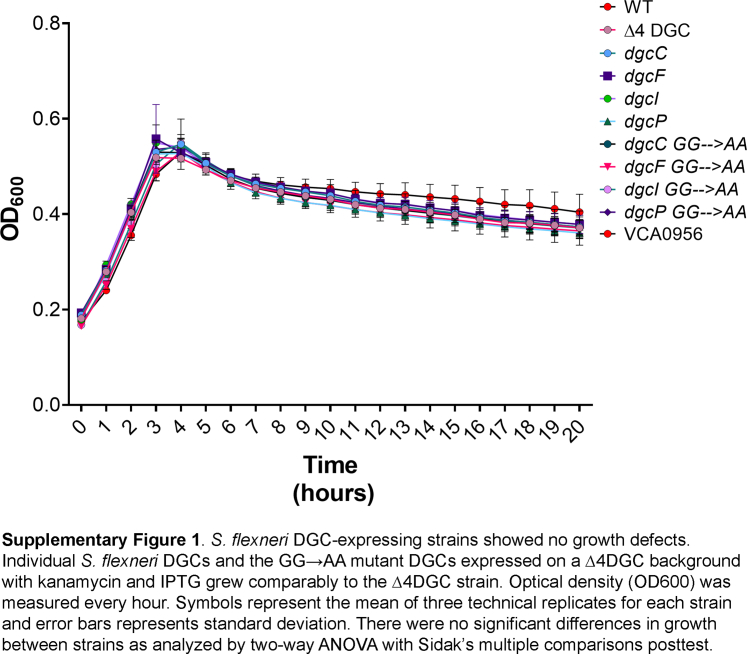


The *S. flexneri* 2457T genome encodes four intact GGDEF domain enzymes, *dgcC* (S0329), *dgcF* (S1698), *dgcI* (S0827), and *dgcP* (S1545) (36). Individual deletion of DGCs from the *S. flexneri* genome significantly reduces biofilm formation and alters invasion, plaque formation, and AR in a DGC-specific manner (20). To study how individual *S. flexneri* DGCs contribute to pathogenesis, we generated a mutant strain where all four intact DGCs (*dgcC*, *dgcF*, *dgcI*, and *dgcP*) were deleted (Δ4DGC). Each of these four *S. flexneri* DGCs were then individually expressed from an IPTG-inducible plasmid in the *S. flexneri* Δ4DGC background. As a negative control, we mutated two amino acids from the active site of each of the four *S. flexneri* DGCs from GG(D/E)EF to AA(D/E)EF (glycine to alanine; GG→AA) to separate the regulatory role of the protein itself to its c-di-GMP synthesis (29). This mutation has been shown to eliminate c-di-GMP synthesis in DGCs from other bacteria (29, 30, 37). We confirmed that the expression of these four DGCs did not alter *S. flexneri* growth in broth (Fig. S1).

**Figure 1:**
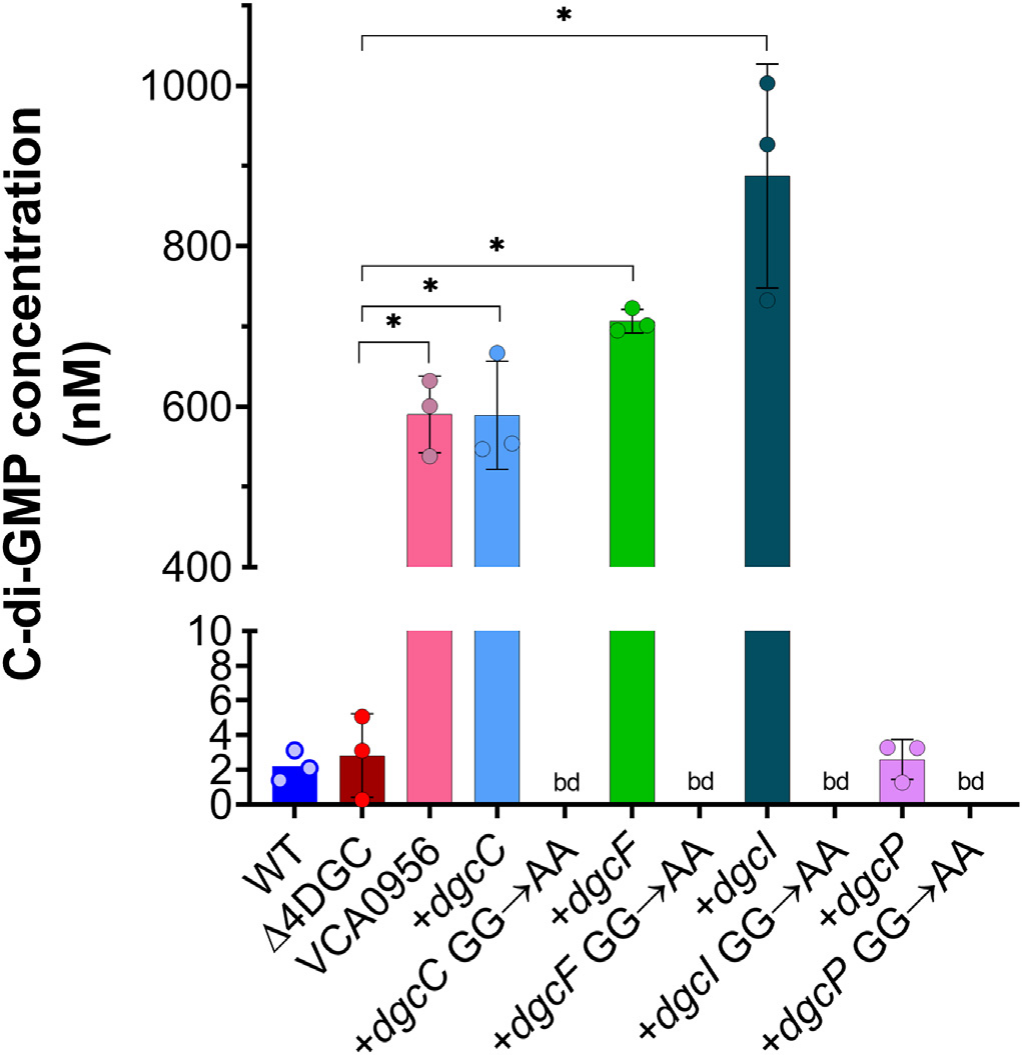
***Shigella flexneri* intact DGCs regulate c-di-GMP levels.** Individual *S. flexneri* DGCs were expressed in the *S. flexneri* Δ4DGC strain, and c-di-GMP levels were measured using LC-MS/MS. *dgcC*, *dgcF*, and *dgcI* expression in the Δ4DGC strain showed significantly more c-di-GMP synthesis than the GG→AA mutants or the empty vector controls, extracted at mid log phase. Expression of *dgcP* did not significantly alter *S. flexneri* c-di-GMP levels in comparison to the Δ4DGC strain. We also noticed no significant difference between WT *S. flexneri* and the Δ4DGC strain. VCA0956 (positive control) significantly increased c-di-GMP levels in *S. flexneri* (20). bd indicates below detection, ∗ represents significant differences between strains as analyzed by one-way ANOVA with Sidak’s multiple comparisons post test (*p < 0.05*). Each symbol represents independent replicates, and error bars indicate SD. c di-GMP, cyclic di-GMP; DGC, diguanylate cyclase.

Ectopic expression of the *Vibrio cholerae* DGC VCA0956 significantly increases c-di-GMP in *S. flexneri* as measured by LC-MS/MS (20). We hypothesized that the expression of intact *S. flexneri* DGCs will increase c-di-GMP levels as compared to GG→AA mutant alleles. C-di-GMP from these strains was extracted during mid log growth (∼3 h post IPTG addition) and quantified using LC-MS/MS. We observed that *dgcI* expression resulted in the highest levels of c-di-GMP, whereas *dgcC*, and *dgcF* expression increased c-di-GMP to levels comparable to the VCA0956 expression strain. Contrary to that, the *dgcP* expression strain showed no difference in c-di-GMP levels to that of the Δ4DGC strain (Fig. 1). C-di-GMP was below our limit of detection when individual GG→AA DGC mutants were expressed, but interestingly, we observed detectable levels of c-di-GMP in the *S. flexneri* Δ4DGC strain carrying an empty plasmid, comparable to the WT strain (Fig. 1).

### *S. flexneri dgcP* expression increases c-di-GMP 3 h postinduction

**Figure 2:**
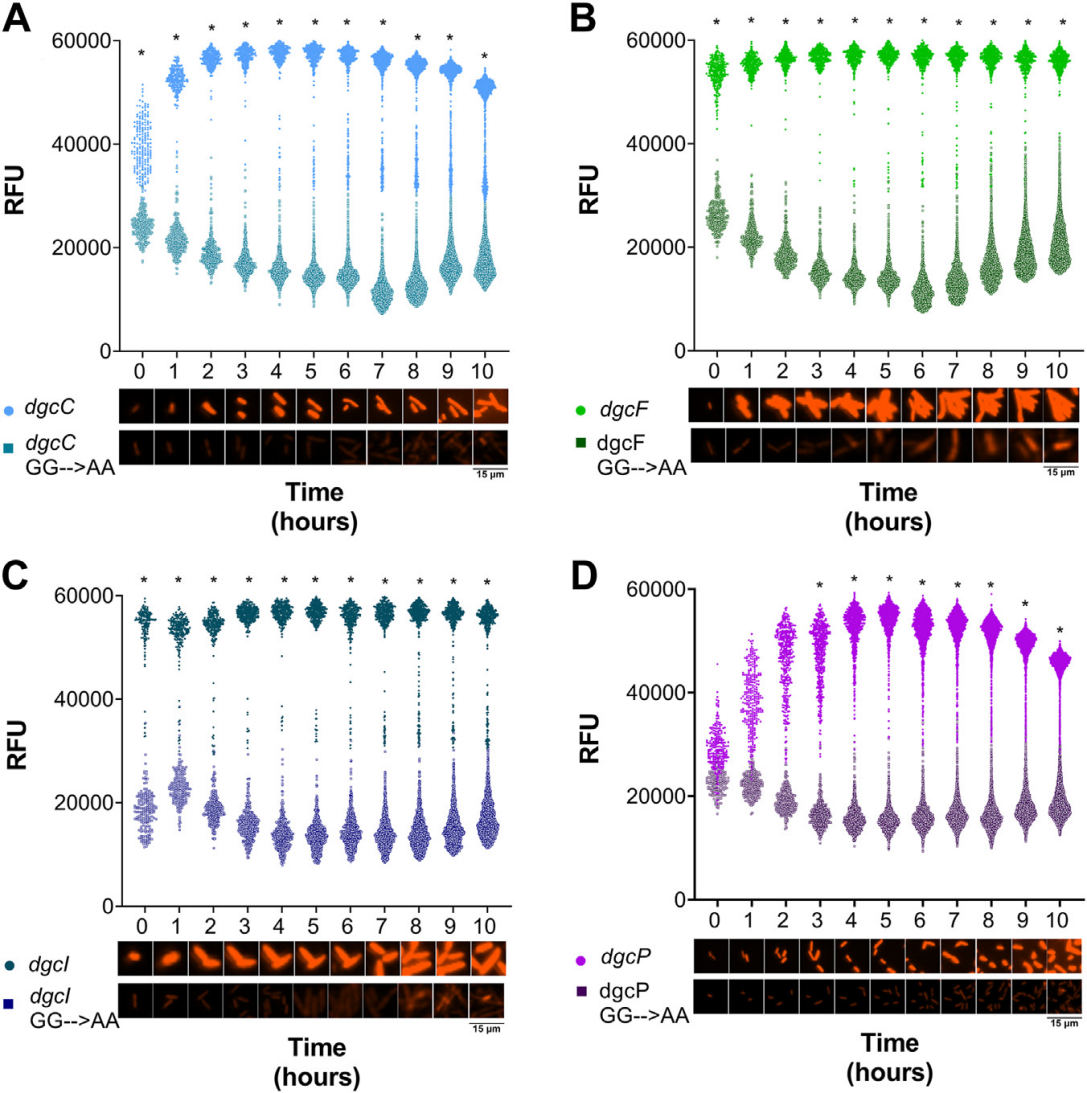
**DGC expression significantly increases c-di-GMP–dependent fluorescence compared to GG→AA mutants.** Single bacterial cell fluorescence (relative fluorescence units, RFU) was measured using microscopy over time for the *Shigella flexneri* Δ4DGC strain expressing (*A*) *dgcC*, (*B*) *dgcF*, (*C*) *dgcI*, and (*D*) *dgcP* (*lighter colored circles*), alongside their respective GG→AA mutants (*darker colored, opensquares*). *S. flexneri* DGCs were expressed from an IPTG-inducible plasmid and a second plasmid expressing the c-di-GMP–specific double tandem riboswitch with m-Scarlett I (riboswitch reporter) was used to measure c-di-GMP–dependent fluorescence. Each of the four DGC expression strains fluoresces more than the GG→AA mutant expression strains. *Symbols* indicate individual fluorescent cells for each time point for each strain. The *graph* is representative of one of the three independent trials. Images below the graph (*A*–*D*) are representative samples of individual bacterial cells; *red* indicates c-di-GMP–dependent m-Scarlett I fluorescence. Significant fluorescence differences between strains were analyzed using two-way ANOVA with Sidak’s multiple comparisons post test (∗*p < 0.05*) at each time point. c di-GMP, cyclic di-GMP; DGC, diguanylate cyclase.

**Figure S2:**
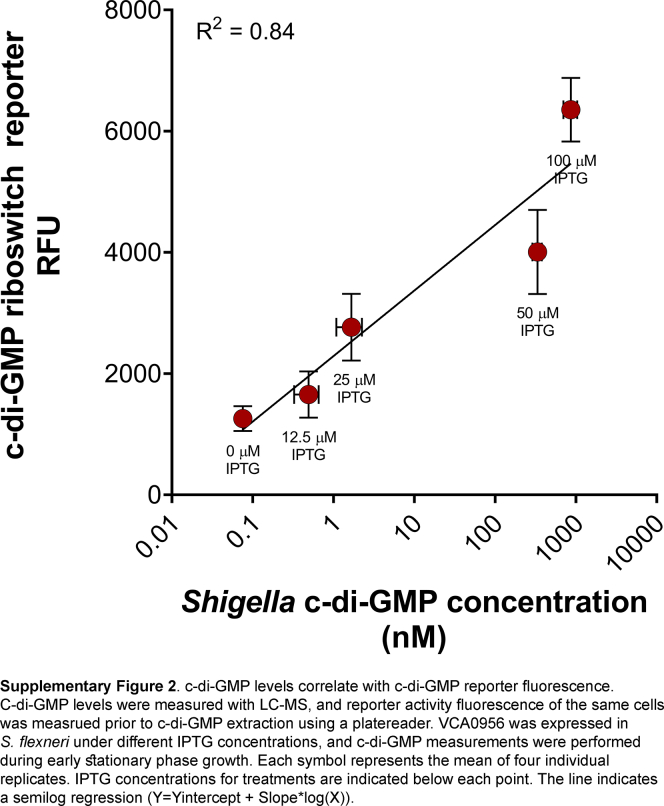


**Figure S3:**
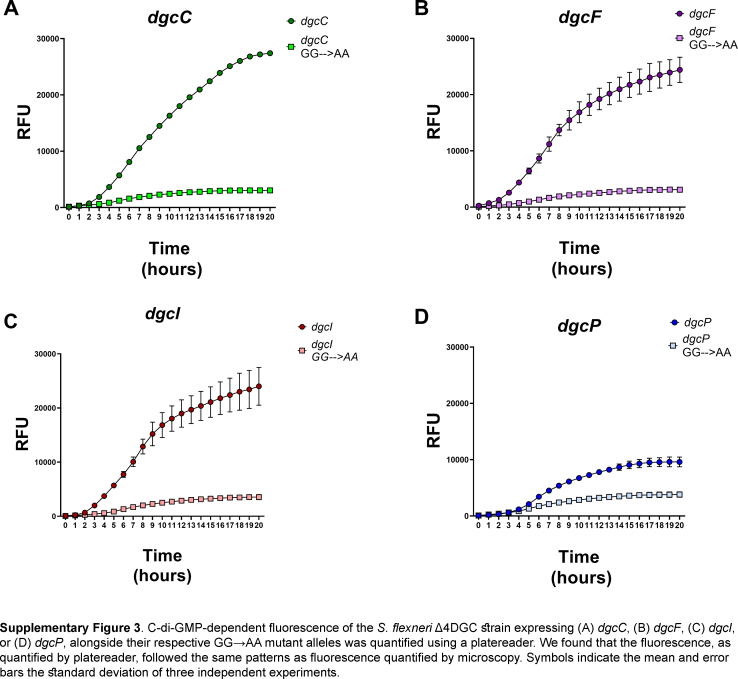


We were intrigued that *S. flexneri dgcP* expression did not increase c-di-GMP levels compared to the other three DGCs (Fig. 1). We further wanted to investigate temporal dynamics of the four intact *S. flexneri* DGCs; thus, as a complementary approach, we quantified relative c-di-GMP levels using a plasmid encoding a double tandem riboswitch Bc3-Bc4 controlling the expression of the reporter gene m-Scarlett I (riboswitch reporter)(38), allowing c-di-GMP measurement with single-cell resolution of live cells by microscopy. The riboswitch reporter binds c-di-GMP with high affinity and specificity, and in the absence of c-di-GMP, a terminator inhibits the translation of m-Scarlett I. This c-di-GMP reporter was validated in the closely related *E. coli* (38). We confirmed that the reporter functions similarly in *S. flexneri*, by comparing our positive control, VCA0956 LC-MS/MS measurements to the c-di-GMP reporter fluorescence from the same bacterial cultures (Fig. S2). We hypothesized that *S. flexneri* DGC expression would result in m-Scarlett I fluorescence that correlates with c-di-GMP levels measured by LC-MS/MS. We quantified m-Scarlett I fluorescence of our *S. flexneri* DGC expression strains over time using live-cell fluorescence microscopy. We then compared the mean of individual fluorescent cells of each DGC to the GG→AA mutants. Of note, we observed no differences in cell size and growth among all our strains. As expected, c-di-GMP–dependent fluorescence increased rapidly and significantly over time in *S. flexneri dgcC*, *dgcF*, and *dgcI*, reaching near maximal fluorescence after 1 h (Fig. 2, *A*–*C*). Interestingly, *S. flexneri dgcP* expression increased fluorescence at a slower rate, resulting in significantly higher c-di-GMP levels 3 h post IPTG induction than the GG→AA *dgcP* mutant (Fig. 2*D*). We observed similar patterns when the fluorescence of these strains was quantified using a plate reader (Fig. S3). We speculate that differences in the growth conditions used for quantitation by LC-MS/MS and microscopy alter *S. flexneri* DgcP c-di-GMP synthesis (Figs. 1 and 2*D*).

### *S. flexneri* c-di-GMP levels are dynamic during host cell growth

**Figure 3:**
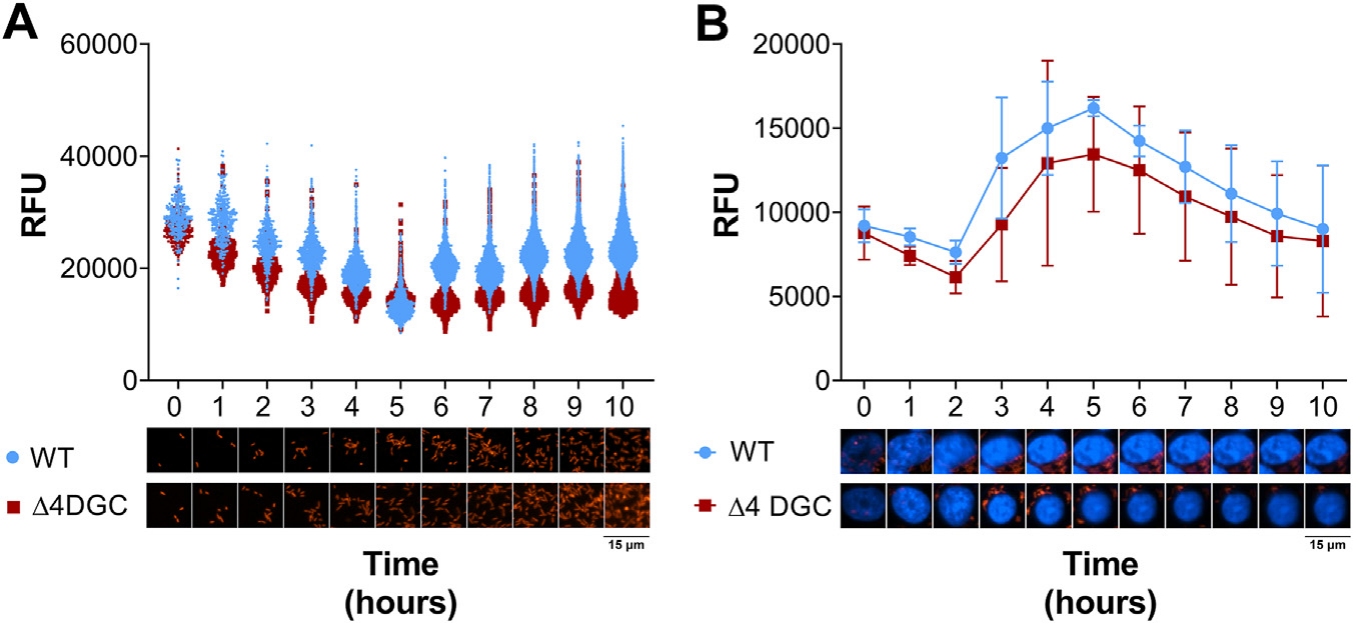
***Shigella flexneri* increases c-di-GMP production *in vivo* 3 h post infection.** c-di-GMP–driven fluorescence (RFU) of *S. flexneri* WT and the Δ4DGC strain carrying the c-di-GMP riboswitch reporter grown (*A*) in culture or (*B*) in Henle-407 cells. *A*, in culture, c-di-GMP reporter fluorescence levels remained relatively constant over the course of 10 h and were consistently but nonsignificantly higher in the WT *S. flexneri* strain than the Δ4DGC strain. *Symbols* indicate the individual fluorescent cells, representative of one trial among the three. *B*, in both the *S. flexneri* WT and Δ4DGC strain, c-di-GMP reporter fluorescence levels increased 3 HPI and then gradually decreased starting at 5 HPI. Images below the graph are representative of individual bacterial cells in culture. Infected Henle-407 cells were stained with Hoechst to label host cell nuclei (*blue*). *Red* indicates c-di-GMP–dependent m-Scarlett I fluorescence. Significant differences between strains were analyzed using two-way ANOVA with Sidak’s multiple comparisons posttest (∗*p < 0.05*). c di-GMP, cyclic di-GMP; DGC, diguanylate cyclase; RFU, relative fluorescence unit.

As *S. flexneri* is an intracellular pathogen, we were interested in c-di-GMP changes during growth in a host cell, and if the four intact *S*. *flexneri* DGCs are responsible for c-di-GMP production in this environment. To answer these questions, we used our riboswitch reporter to evaluate changes in c-di-GMP production of live *S. flexneri* cells over time. When WT *S. flexneri* was grown in a defined medium, c-di-GMP remained relatively constant, exhibiting modestly decreasing fluorescence over time, although this trend was not statistically significant. The *S. flexneri* Δ4DGC strain was consistently lower than the WT at all time points, although this difference too was not statistically significant (Fig. 3*A*).

We performed the same analysis to assess changing c-di-GMP reporter fluorescence over time during *S. flexneri* infection of Henle-407 cells (Fig. 3*B*)(39). In contrast to *S. flexneri* grown in culture, WT *S. flexneri* fluorescence increased at 3 h postinfection (HPI), peaked 5 HPI, and then gradually decreased fluorescence during Henle-407 infection. For the *S. flexneri* Δ4DGC strain, we again observed that c-di-GMP reporter fluorescence levels were consistently nonsignificantly lower than in the WT strain, but we saw the same general pattern, where fluorescence increased at 3 HPI, peaked at 5 HPI, and then decreased afterward (Fig. 3*B*).

### *S*. *flexneri* DGCs produce c-di-GMP during host cell infection

**Figure 4:**
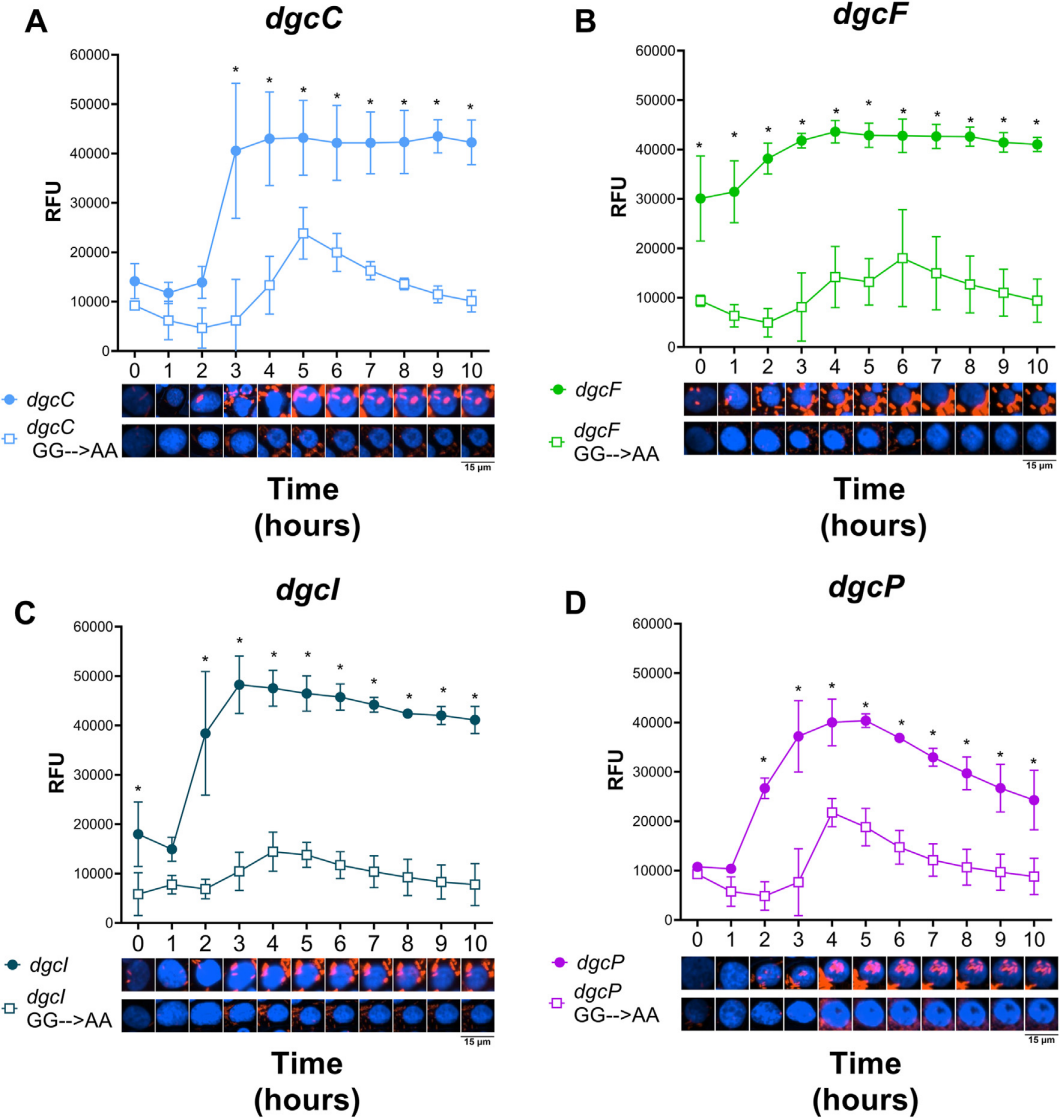
***Shigella flexneri* DGCs regulate c-di-GMP synthesis post infection.** C-di-GMP–dependent fluorescence (RFU) of the *S. flexneri* Δ4DGC strain expressing (*A*) *dgcC*, (*B*) *dgcF*, (*C*) *dgcI*, or (*D*) *dgcP*, alongside their respective GG→AA mutant alleles, was quantified during infection of Henle-407 cells. Expression of all four intact DGCs significantly increased fluorescence compared to individual GG→AA mutant DGCs, but the time it took for these differences to become significant was variable. Representative micrographs below illustrate single-cell fluorescence; *red* indicates c-di-GMP–dependent m-Scarlett I fluorescence, and blue host cell nuclei stained with Hoechst. *Symbols* indicate the mean fluorescence of single-cell measurements from three independent replicates, and error bars indicate SD. Significant differences between strains at each time point were analyzed using two-way ANOVA with Sidak’s multiple comparisons posttest (∗*p* < 0.05). c di-GMP, cyclic di-GMP; DGC, diguanylate cyclase; RFU, relative fluorescence unit.

To determine how *S. flexneri* DGCs contribute to changing c-di-GMP levels during host cell growth, we compared the expression of *S. flexneri dgcC*, *dgcF*, *dgcI*, *dgcP*, and their respective GG→AA mutants in the *S. flexneri* Δ4DGC strain during infection. Expression of each of the four intact DGCs increased c-di-GMP reporter fluorescence, but the temporal dynamics were different for each strain (Fig. 4). Specifically, the time that it took each DGC to initiate c-di-GMP synthesis varied. *dgcC* expression exhibited no significant fluorescence until 3 HPI and then increased rapidly (Fig. 4*A*). *dgcF* expression resulted in constitutively high c-di-GMP reporter fluorescence throughout the entire course of infection (Fig. 4*B*). *dgcI* expression did not increase fluorescence until 2 HPI (Fig. 4*C*). Similar to *dgcI*, *dgcP* expression increased starting at 2 HPI and peaked at 5 HPI and then steadily decreased for the remainder of the experiment (Fig. 4*D*). Interestingly, for each of our GG→AA mutants, we observed an increase in fluorescence that peaked between 4 and 6 HPI and then decreased as the infection progressed.

### *dgcF* expression increases *S. flexneri* acid sensitivity in AR1-inducing conditions

**Figure 5:**
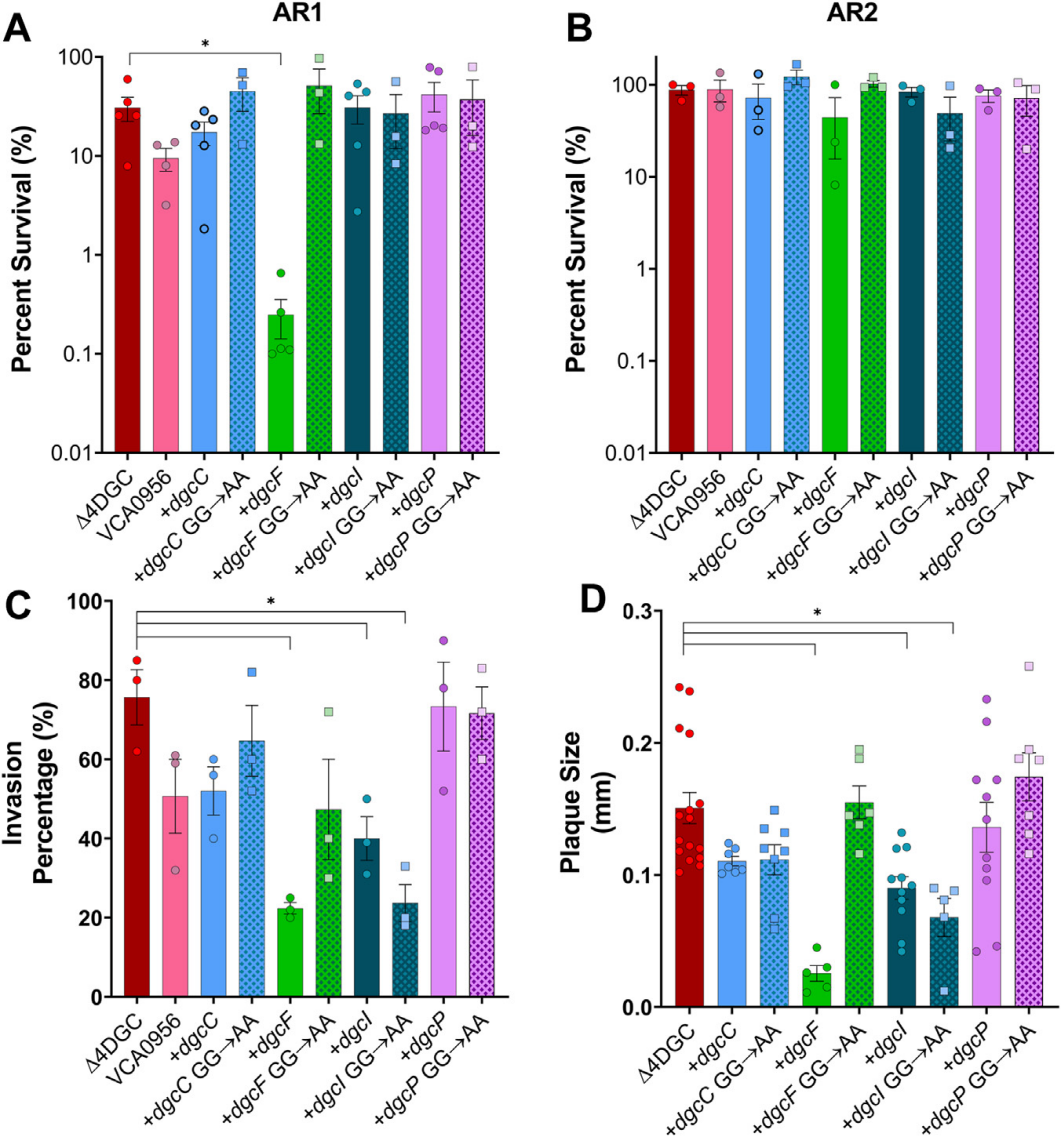
**Expression of *Shigella flexneri dgcF* increases acid sensitivity in AR1-inducing conditions and reduces virulence.***A*, each *S. flexneri* intact DGC was expressed in the Δ4DGC strain and challenged in pH 2.5 medium in AR1-inducing conditions for 1 h. Percent survival was determined by quantifying the subsequent CFU/ml of cultures challenged in pH 2.5 medium, compared to organisms in pH 7.0 medium. *dgcF* expression significantly decreased acid resistance as compared to the *S. flexneri* Δ4DGC strain carrying an empty plasmid and the GG→AA mutant *dgcF*, as determined by a Kruskal–Wallis test with Dunn’s multiple comparisons post test. *B*, the same experiment was performed in AR2-inducing conditions, which relies on external glutamate (41, 42). AR2-inducing conditions resulted in overall higher *S. flexneri* survival, and we observed no significant differences in survival when each intact DGC or GG→AA mutant was expressed. *Bars* represent the mean of three independent trials, and error represents SD. *C*, we assessed the capacity of *S. flexneri* strains to invade Henle-407 cells. *dgcF*, *dgcI*, and the *dgcI* GG→AA mutant expression in the Δ4DGC strain show significant reduction in the percentage of invaded cells. *Bars* indicate the mean of three independent trials, and error represents SD. *D*, plaque size was measured after infecting Henle-407 cells with the *S. flexneri* Δ4DGC strain expressing the four intact DGCs or GG→AA mutant alleles. *dgcF*, *dgcI*, and the GG→AA mutant *dgcI* strains show significant reduction in plaque size as compared to the *S. flexneri* Δ4DGC mutant strain carrying an empty plasmid. *Symbols* represent individual plaques from one replicate among the three individual trials. *Bars* indicate the mean, and error bars represents the SD. Significant differences were analyzed using two-way ANOVA with Sidak’s multiple comparisons posttest (∗*p < 0.05*). AR, acid resistance; c di-GMP, cyclic di-GMP; DGC, diguanylate cyclase.

During human infection, *Shigella* must survive the highly acidic environment in the stomach before it can access the human colon. *Shigella* survives acid stress with at least two systems (AR1 and AR2) that are induced in different conditions (40, 41, 42, 43, 44). We previously showed that deletion of *S. flexneri dgcF* increases AR (20); therefore, we hypothesized that expression of *S. flexneri dgcF* will decrease AR. We preconditioned *S. flexneri* DGCs in complex media at pH 5.5 to induce AR1 and separately at pH 5.5 with glucose and low oxygen to induce AR2. We then challenged our DGC expression strains with exposure to defined media at pH 2.5 (to study AR1) or pH 2.5 with glutamate (to study AR2) (40, 41, 42, 45). *dgcF* expression significantly reduced acid survival in AR1-inducing conditions, compared to other DGCs and the GG→AA mutants (Fig. 5*A*). In AR2-inducing conditions, DGC expression did not significantly decrease AR (Fig. 5*B*). This suggests that *S. flexneri dgcF* specifically regulates acid survival in AR1-inducing conditions.

### Virulence phenotypes in *S. flexneri* are regulated by *dgcF and dgcI*

VCA0956 expression reduces *S. flexneri* invasion and plaque formation, but the *S. flexneri* Δ*dgcF* strain also has reduced invasion and forms smaller plaques in Henle-407 monolayers (20). To see how expressing the four intact *S. flexneri* DGCs impacts these phenotypes, we examined the invasion and plaque phenotypes affected by our DGC expression strains. Henle-407 cells were infected with the *S. flexneri* Δ4DGC strain with DGC expression plasmids and their respective GG→AA mutants. As a control, we included VCA0956 expression which decreases invasion (20). Expression of *dgcF* and *dgcI* exhibited invasion defects as compared to the empty plasmid control, while *dgcC* and *dgcP* expression strains show no significant difference in invasion (Fig. 5*C*). Interestingly, the *dgcI* GG→AA mutant also exhibited an invasion defect, while all other GG→AA DGC mutants did not.

We also examined the ability of each DGC expression strain to form plaques in cell culture monolayers, as previously described (39). Each strain was induced 30 min postinfection to ensure comparable invasion. Similar to the invasion assay, we found that the *dgcF*, *dgcI*, and *dgcI* GG→AA mutant showed reduced plaque size in comparison to the empty plasmid control, while *dgcC* and *dgcP* expression showed no significant difference in plaque size compared to the Δ4DGC (Fig. 5*D*).

### Four *S. flexneri* DGC pseudogenes encode GGDEF domains after nonsense mutations

**Figure S4:**
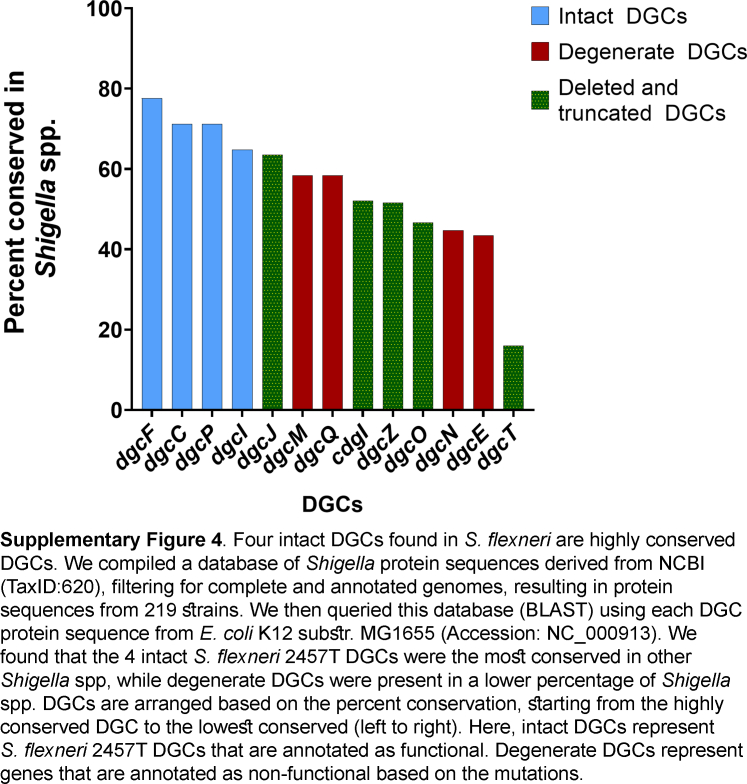


Our LC-MS/MS measurements and riboswitch reporter data indicate that the *S. flexneri* Δ4DGC strain still produces c-di-GMP; therefore, we sought to identify other *S. flexneri* genes that synthesize c-di-GMP. We started by investigating the other DGC pseudogenes. In comparison to *E.coli* K12 substr. MG1655 (Accession: NC_000913), four GGDEF domain and one GGDEF-EAL domain genes are annotated as pseudogenes (considered degenerate) in *S. flexneri* 2457T (Accession: AE014073.1) and include deletions, frameshift mutations, and/or nonsense mutations that presumably disrupt the GGDEF domain (20, 36). Compared to *E. coli*, there are nine other DGCs that either do not contain the GGDEF domain or are deleted in *S. flexneri* (36). Therefore, for the scope of this study, we focused on the 5 GGDEF and GGDEF-EAL domain encoding genes with premature stop codons (Table 1). Recent studies indicate that other *S. flexneri* degenerate enzymes have demonstrable phenotypes (46); therefore, we hypothesized that one or more of these DGC pseudogenes retain c-di-GMP synthesis. Compared to *E. coli* MG1655, *dgcT*, *dgcZ* and *cdgI* genes are completely absent from the *S. flexneri* 2457T genome (Table 1). *S. flexneri dgcO* (S1870) and *dgcJ* (S1553) genes have truncating deletions that remove the GGDEF domain. The remaining four *S. flexneri* genes, *dgcM* (S1445), *dgcN* (S2841), *dgcE* (S2255), and *dgcQ* (S2095), have pairwise identity >97.9% compared to *E. coli* K12 strain; however, each of these genes encodes a nonsense mutation resulting in a stop codon preceding the GGDEF domain, which we confirmed by whole genome sequencing. Notably, these four DGC genes have alternative reading frames that begin close to the nonsense mutation site and encode an intact GG[D/E]EF domain. *S. flexneri dgcM*, *dgcE*, and *dgcQ* all contain an RXXD inhibition site, potentially enabling allosteric feedback inhibition (Table 1) (47, 48). Interestingly, while the four intact *S. flexneri* 2475T DGCs are the most well conserved in other *Shigella* spp., degenerate DGCs are intact in the genomes of other *Shigella* spp. (Fig. S4).

**Table 1:** *Shigella* degenerate and deleted DGCs

Gene	Locus	Other names	GGDEF domain			Mutation	Predicted size
			I-site		A-site		
*dgcE*	S2255	*yegE*	RSSD	VLARL	GGDEF	Premature Stop codon at 366 AA; MET at 330	46.37 kDa and 70.79 kDa
*dgcJ*	S1553	*yeaJ*				Truncating deletion, no GGDEF	
*dgcM*	S1445	*ydaM*	RKGD	LVFRW	GGEEF	Premature Stop codon at 177 AA; MET at 180	23.88 kDa and 27.16 kDa
*dgcN*	S2841	*yfiN*	GLRH	KAYRL	GGDEF	Premature Stop codon at 120 AA; MET at 101	17.23 kDa and 36.4 kDa
*dgcO*	S1870	*yddV, dosC*				Truncating deletion, no GGDEF	
*dgcQ*	S2095	*yedQ*	RAQD	VAGRV	GGEEF	Premature Stop codon at 56 AA; MET at 59	10.19 kDa and 58.7 kDa
*dgcT*		*ycdT*				Deletion	
*dgcZ*		*ydeH*				Deletion	
*cdgI*		*yeaI*				Deletion	
*pdeA*	S2600	*yfeA*	QENE	KLYQL	PGSEL		
*pdeF*	S2699	*yfgF*	EPGE	DVYQL	SGNDL		
*pdeK*	S4206	yhjK	SPRM	ILAQI	SGYDF		
*pdeO*	S1871	*yddU, dosP*	KPDQ	YLCRI	EGTQF		
*pdeR*	S1372	*yciR*	EHDQ	VLARP	GGDEF	Premature Stop codon at 154 AA; MET at 161	21.01 kDa, 32.20 kDa and 26.46 kDa

*Shigella flexneri* 2457T encodes 14 degenerate DGCs containing mutations that disrupt the GGDEF domain. To retain predicted function, the gene should encode GG[D/E]EF in the active site (A-site). The presence of RXXD (underlined) in the I-site indicates allosteric regulation. Five genes encode premature stop codons preceding the GGDEF domain, five genes include deletions that have removed the GGDEF domain, and four genes encode GGDEF domains without A-site residues required for c-di-GMP synthesis. Mutations were confirmed by whole-genome sequencing (Plasmidsaurus). The predicted protein size of genes encoding a premature stop and an alternative reading frame include a 6× His-tag.

DGC, diguanylate cyclase.

### *S. flexneri dgcE* and *dgcQ* produce c-di-GMP

**Figure 6:**
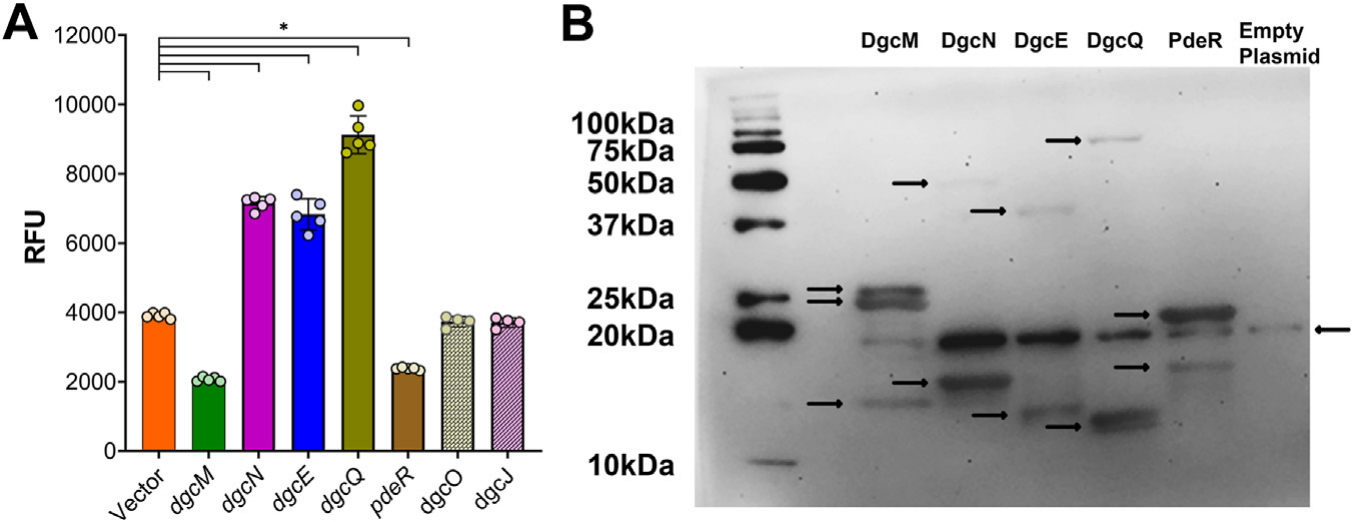
***Shigella flexneri dgcN, dgcE,* and *dgcQ* significantly increase c-di-GMP levels.***A*, *S. flexneri* degenerate DGCs were expressed in *Escherichia coli* BL21 and c-di-GMP levels measured using the c-di-GMP riboswitch reporter. *dgcE*, *dgcQ*, and *dgcN* had significantly increased fluorescence in comparison to our empty plasmid control, whereas *dgcM* and *pdeR* showed significantly lower fluorescence. *Individual symbols* represent three independent replicates; ∗ represents significant differences in comparison to the empty plasmid control as determined by one-way ANOVA with Sidak’s multiple comparisons posttest (*p* < 0.05). Error bars indicate SD. *B*, *S. flexneri* degenerate DGCs with both N- and C-terminal 6× His-tags were expressed in *E. coli* BL21, and protein expression was visualized by SDS-PAGE followed by Western blot. Samples for DgcN and DgcE were loaded 2× to visualize faint bands. *Arrows* highlight fragments for each gene expressed. We observed a nonspecific band at ∼20 kDa in all lanes including our empty vector control. c di-GMP, cyclic di-GMP; DGC, diguanylate cyclase.

**Figure S5:**
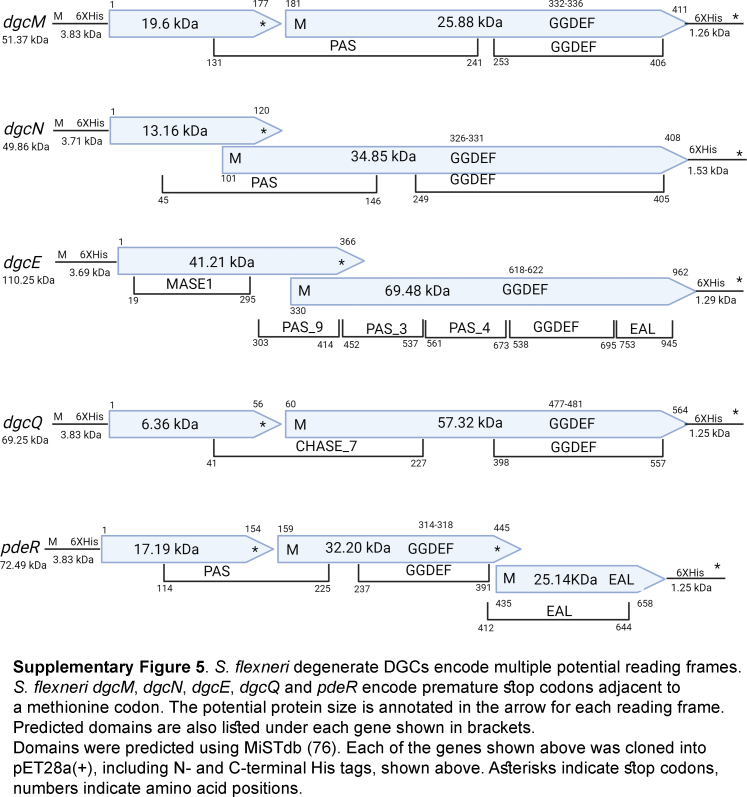


**Figure S6:**
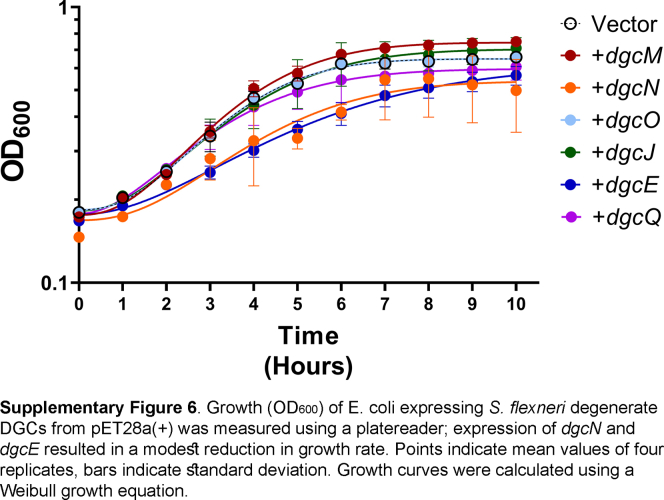


To determine if *S. flexneri* degenerate DGCs are capable of c-di-GMP synthesis, we focused on the five degenerate DGCs that contain nonsense mutations preceding the GGDEF domain. We also included *dgcO* and *dgcJ*, with the expectation that these genes could not produce c-di-GMP due to the complete lack of GGDEF domain sequence. We expressed each of these 7 *S. flexneri* genes (the start codon to the orthologous *E. coli* stop codon) from a high expression plasmid, pet28a(+), with both N- and C-terminal 6X-His-tags in *E. coli* BL21 (Fig. S5). We chose this expression platform to increase the likelihood of observing potentially small amounts of c-di-GMP production from these gene products. We assessed c-di-GMP reporter fluorescence levels using our c-di-GMP riboswitch reporter (Fig. 6*A*). Surprisingly, *dgcE*, *dgcQ*, and *dgcN* expression resulted in fluorescence significantly higher than the empty plasmid control and other degenerate DGCs at 5 h post IPTG induction, consistent with c-di-GMP synthesis (Fig. 6*A*). *dgcM* and *pdeR* expression reduced fluorescence compared to the empty vector control. Expectedly, *dgcO* and *dgcJ* expression did not result in any increase in fluorescence relative to the empty plasmid control. Of note, we observed a modest reduction in growth upon *dgcE* and *dgcN* expression (Fig. S6). This growth inhibition could be due to stress associated with high expression of these gene products or from c-di-GMP production, as *dgcN* expression inhibits *E. coli* growth dependent on c-di-GMP synthesis (49, 50).

We next assessed the protein expression of these five degenerate DGCs by Western blot, using the same plasmids with both N- and C-terminal 6× His-tags. Of note, a nonspecific band was observed at ∼20 kDa in all samples including our empty vector control (Fig. 6*B*). *dgcM* and *dgcQ* expression produced two proteins each, corresponding to their premature stop codon and adjacent methionine (Fig. 6*B*), presumably translating the GGDEF domain in second reading frame (Table 1). *dgcM* expression also produced a third band at ∼15 kDa, which does not match any reading frames. *dgcN* expression produced two bands at ∼17 kDa and ∼40 kDa. The 17 kDa size corresponds to the first N-terminal reading frame; however, the 40 kDa size corresponds with full-length DgcN. *dgcE* expression produced two bands at ∼45 kDa and 15 kDa. The 45 kDa corresponds to the N-terminal reading frame; however, the other band does not correspond with any potential *dgcE* reading frames. *E. coli* DgcE undergoes posttranslational proteolysis, thus it is possible that this processing is why the sizes we observed here did not match the predicted sizes (34, 51). *pdeR* expression also produced two bands; the first band at ∼18 kDa corresponds to the first reading frame, however the second band at ∼22 kDa does not match any predicted reading frames.

### *dgcE* deletion reduces *S. flexneri* c-di-GMP levels

**Figure 7:**
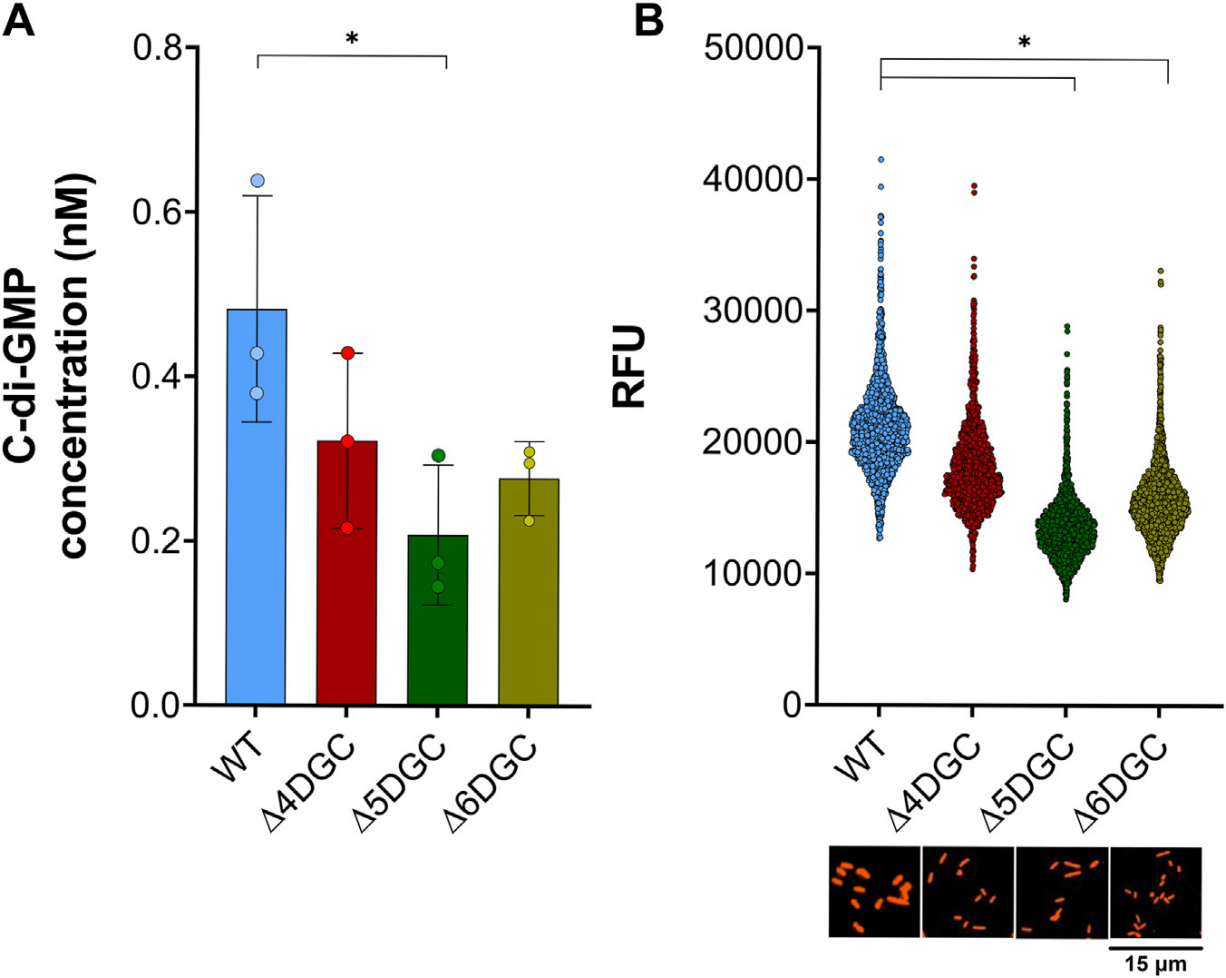
***Shigella flexneri* Δ5DGC and Δ6DGC strains have significantly reduced c-di-GMP levels.***A*, *S. flexneri* Δ5DGC (Δ4DGC + Δ*dgcE*) and Δ6DGC (Δ4DGC + Δ*dgcE* + Δ*dgcQ*) c-di-GMP levels were compared to WT *S. flexneri* using LC-MS/MS. The *S. flexneri* Δ5DGC strain showed significantly lower c-di-GMP levels than the WT *S. flexneri* strain. In the *S. flexneri* Δ6DGC strain, c-di-GMP levels were reduced but not statistically significant than in the WT strain. *B*, *S. flexneri* Δ5DGC and Δ6DGC strain c-di-GMP levels quantified using the c-di-GMP riboswitch reporter. C-di-GMP levels were significantly lower for the Δ5DGC or Δ6DGC strain than WT *S. flexneri*. Each symbol represents individual fluorescent cells for each strain. Images below the graph are individual fluorescent cells for each strain. *Asterisks* represent significant differences among strains as compared using one-way ANOVA with Sidak’s multiple comparisons post test (*p* < 0.05), error bars indicate SD. c di-GMP, cyclic di-GMP; DGC, diguanylate cyclase.

As expression of *S. flexneri dgcE, dgcQ,* and *dgcN* pseudogenes in *E. coli* BL-21 significantly increased c-di-GMP levels, we hypothesized that degenerate DGCs also contribute to c-di-GMP production in *S. flexneri*. To test this hypothesis, we first deleted *dgcE* from the *S. flexneri* Δ4DGC background and generated a Δ5DGC strain (Δ4DGC + Δ*dgcE*). Then, we deleted *dgcQ* in the *S. flexneri* Δ5DGC background, to generate Δ6DGC (Δ4DGC + Δ*dgcE +* Δ*dgcQ*). We quantified the c-di-GMP levels of each of these strains using LC-MS/MS (Fig. 7*A*). The *S. flexneri* Δ5DGC strain exhibited significantly reduced c-di-GMP levels compared to the WT *S. flexneri* strain; however, our Δ6DGC strain showed no significant difference compared to WT *S. flexneri* (Fig. 7*A*). Notably, we still detected a c-di-GMP peak in the *S. flexneri* Δ6DGC strain, indicating that *dgcN* (or other DGCs) may also produce c-di-GMP (Fig. S7). We confirmed our findings using the riboswitch reporter and fluorescence microscopy. Measurements using the riboswitch reporter mirrored those taken by LC-MS/MS, and we observed significant reductions in c-di-GMP reporter activity in both the Δ5DGC and Δ6DGC strains compared to the WT *S. flexneri* strain (Fig. 7*B*).

## Discussion

Because of the evolutionary relationship between commensal *E. coli* and pathogenic *S. flexneri*, we can use these two organisms as a model to examine how c-di-GMP signaling changes in the process of pathoadaptation (52). Only the four DGCs most conserved in other *Shigella* spp. (Fig. S4) have been retained with high fidelity in *S. flexneri* 2457T. One possible reason that these enzymes were conserved over others is signaling specificity, where specific DGCs alter c-di-GMP synthesis and associated phenotypes (31, 33, 34). Our data support this hypothesis, as there is a poor correlation between the expression of each of the four intact *S. flexneri* DGCs and the phenotypes we measured in this study. For example, *dgcF* expression produces less c-di-GMP than *dgcI* expression in *S. flexneri*, but it is the only DGC that significantly decreases acid sensitivity in AR1-inducing conditions. Our observation that *S. flexneri* GG→AA mutated *dgcI* regulated invasion and plaque phenotypes irrespective of catalytic activity is also suggestive that protein–protein interactions are enabling specific phenotypes (24, 53, 54). An alternative explanation regarding the discrepancy between measured c-di-GMP and various phenotypes resulting from DGC expression is that environmental regulation is contributing to differences we observe between c-di-GMP measurements and phenotypes, as environmental conditions in phenotypic assays do not precisely match those used for c-di-GMP measurements, and DGCs recognize environmental signals *via* their variable N-terminal domains that alter enzymatic activity at the C-terminal domain (33, 55). One limitation of this study is that although these genes are being expressed from the same promoter in these experiments, it is also possible that differences in protein expression could be contributing to c-di-GMP–associated differences we observe here.

We observed that c-di-GMP reporter fluorescence levels inside host cells were dynamic during infection (Fig. 3), and that *S. flexneri* DGCs produced c-di-GMP differentially in this environment (Fig. 4). *S. flexneri* virulence gene expression is dynamic during growth in an epithelial cell, and the timing correlates with the increase in c-di-GMP we observed (10, 53, 56, 57, 58). Expression of VCA0956 in *S. flexneri*, which raises c-di-GMP, significantly downregulates the expression of virulence genes, including those of the type III secretion system (20), and a similar phenomenon was also reported in *Salmonella typhimurium* (24). Thus, it is possible that c-di-GMP is contributing to differential gene expression during growth in a host cell, but the signal transduction pathway linking c-di-GMP signaling and virulence gene regulation is still unknown.

Surprisingly, we found that deleting all four intact DGCs from the *S. flexneri* genome reduced c-di-GMP levels but did not eliminate c-di-GMP and that expression of *S. flexneri* degenerate DGCs *dgcE*, *dgcQ*, and *dgcN* increased c-di-GMP (Fig. 6). The degenerate DGCs in *S. flexneri* are among those that synthesize c-di-GMP in other *Escherichia*/*Shigella* spp (21, 59). Expression of these *S. flexneri* degenerate DGC genes results in multiple protein isoforms, some of which could potentially contain the GGDEF domain (Fig. 6*B*). *E. coli dgcE* is a large protein, encoding ten transmembrane domains within the first 300 residues. It also encodes a predicted MASE1 domain and three PAS domains before a GGDEF and degenerate EAL domain. *E. coli* DgcE undergoes rapid proteolysis through an unknown protease, resulting in protein degradation. However, this pattern was not observed in the ΔMASE (N terminal) DgcE, suggestive of proteolysis initiating from the N-terminal domain to the C-terminal domain (34, 51). DgcE initiates the expression of biofilm regulator CsgD and mediates curli and cellulose production in *E. coli*, facilitating exposure to host immune system (60, 61, 62, 63). *S. flexneri dgcE* contains IS110 family transposase insertion, resulting in a premature stop codon and a frame shift; nevertheless, we found that deletion of *dgcE* from the Δ4DGC background significantly reduced c-di-GMP. *dgcE* has been annotated as a pseudogene in nine other pathogenic *E. coli* strains, including EHEC, EPEC, and EAEC isolates, suggesting a broader evolutionary adaptation associated with pathogenic host colonization (64).

In the case of *dgcQ* and *dgcN*, expression of these genes corresponded with two proteins each, one matching the size of the N-terminal reading frame and the other the full-length protein if it had no stop codon. It is unclear how *S. flexneri* is producing a full-length protein when both of these genes have nonsense mutations. One possibility is translational readthrough (65, 66, 67, 68, 69), or alternatively the bacteria could be inserting selenocysteine into these proteins *in lieu* of the stop codon. *dgcQ* mutations have been observed in at least six different strains of EAEC in the same specific pattern observed in *S. flexneri* 2457T, with an insertion of stop codon at 312 amino acid position and adjacent methionine (AUG) at 314 codon; this suggests that, like *dgcE*, the *dgcQ* adaptation is not unique to this specific *Shigella* strain (64).

*S. flexneri dgcM* expression in *E. coli* reduced c-di-GMP levels and was also expressed in two sizes, but these sizes corresponded to the N-terminal and C-terminal portions being separately translated. If one of these two proteins is the DgcM C-terminal portion, it is not clear how they are expressed without a ribosomal binding site. While *dgcM* does not contain a canonical Shine Dalgarno motif prior to the second start codon, it does contain an A-rich sequence upstream of the second reading frame (AAATAAAT), which can be sufficient to initiate translation (70). Alternatively, *dgcM* could be translating the second reading frame through translational coupling, where the ribosome translates an adjacent gene, modulated by the gene upstream to it (71, 72).

The mutations in *S. flexneri dgcQ*, *dgcE*, and other degenerate DGCs exhibit a very similar pattern, suggesting that this type of mutation is a wider evolutionary mechanism to alter the activity of this enzyme. Deletion of *dgcE* and *dgcQ* from the *S. flexneri* Δ4DGC strain still did not eliminate c-di-GMP in the cell, indicating that other DGCs are still synthesizing the second messenger. It is possible that *dgcN* or *dgcM* are capable of c-di-GMP synthesis when expressed in *S. flexneri*, or the *pdeR* gene (containing both an EAL and GGDEF domain) has a premature nonsense mutation preceding a GGDEF domain, similar to *dgcE* and *dgcQ*.

## Experimental procedures

### Strain construction and plasmids

*Shigella* strains used in this study were routinely cultured on tryptic soy broth agar with Congo red, and red colonies were selected to maintain the virulence plasmid (73). Antibiotic concentrations used were as follows until unless otherwise specified: 25 mg/ml ampicillin, 50 mg/ml chloramphenicol, 20 mg/ml gentamicin, 50 mg/ml kanamycin, and IPTG was added at 100 μM. Other details of strains used are summarized in Table S1. *S. flexneri* Δ4DGC, Δ5DGC, and Δ6DGC mutant strains were generated using homologous recombination (74). Briefly, single genes were deleted by insertion of the *cam* gene flanked by FRT sites, which were later removed by expressing flippase. DGC expression plasmids were constructed by restriction cloning, using the IPTG inducible pEVS143 (75) as the parental plasmid. The GG→AA mutants were generated by site-directed mutagenesis, using Phusion polymerase (NEB). All strains and plasmids were verified using sequencing. All primers used for homologous recombination and plasmid cloning are listed in Table S2. For infection assays, we used human Henle-407 cells (ATCC CCL-6, HeLa contamination line) as previously described (39).

### C-di-GMP quantification using LC-MS/MS method

Overnight cultures from single colonies were grown in LB media with appropriate antibiotics at 30 °C with shaking. Next day, the overnight cultures were subcultured 1:100 in freshly prepared M63 media with Kan and IPTG. The absorbance (*A*_600_) of the subcultures grown at 37 °C for ∼3 h was recorded. From this point, the cultures were centrifuged at 4 °C for 5 min. The supernatant was removed and the pellet was resuspended in 500 μl ice cold extraction buffer (40% acetonitrile, 40% methanol, and 0.1 N formic acid). The resuspended pellet was incubated at −20 °C for 30 min and then centrifuged for 20 min at 4 °C. After centrifugation, the liquid was collected and stored at −80 °C until analysis. Prior to analysis, samples were dried using a vacuum concentrator and then rehydrated in 100 μl water. Quantification of c-di-GMP was performed using a Quattro Premier XE mass spectrometer (Waters) coupled with an Acquity high performance liquid chromatography system (Waters) at the Western Michigan University Homer Stryker M.D. School of Medicine (WMeD), at mass 690.69→344.3. Using microscopy, we estimated the mean volume (2.36 × 10^−15^ L) of *S. flexneri* cells, which was used to determine the intracellular c-di-GMP. The intracellular c-di-GMP concentrations were determined by dividing the c-di-GMP extracted for each strain by the estimated mean volume. Total cell volume of extracted bacteria was calculated by multiplying the CFU/ml to the volume of one bacterium. Experiments were performed in three individual replicates. Graphs were plotted using GraphPad prism (https://www.graphpad.com).

### Microscopy for c-di-GMP quantification using riboswitch reporter

*S. flexneri* strains containing DGC expression plasmids and the biosensor plasmid were grown in LB media with Kan and Amp at 30 °C for ∼16 h. Next day, the strains were subcultured in M63 media with Kan and Amp and were grown for ∼3.5 h. After incubation, the cultures were diluted 1:1000 in M63 media with Kan, Amp, and IPTG. Immediately, 100 μl of this suspension was transferred to a glass-bottom 96-well plate, centrifuged for 10 min at 1000*g*, and cells were imaged using a BioTek Lionheart FX automated fluorescence microscope for 10 h, with half hour interval. Samples were incubated at 37 °C during imaging. Time zero indicates the first microscopy read after adding IPTG and 10 min centrifugation. Our positive control, the VCA0956 expression strain, was used to optimize the tetramethylrhodamine channel exposure. We used autofocus with a 25 μM Z stack projection and beacons were defined for each well. Fluorescence quantitation was performed using BioTek Gen5 software (https://www.agilent.com/), using fluorescence autothreshold and size threshold between 2 μM and 20 μM. An average cell count for each strain was ∼400 cells at time 0 and ∼4000 cells at the 10th hour for each strain. Experimental quantitation of fluorescence was performed three independent times.

### Host cell c-di-GMP kinetics read with microscopy

Henle-407 cells were split in a glass-bottom 96-well plate 2 days before experiment to gain ∼80% confluency (1:3 dilution). One day before experiment, *S. flexneri* cultures were grown with Kan and Amp in LB media at 30 °C for ∼16 h. On the day of experiment, all the strains were subcultured in M63 media with Kan, Amp, and DOC (0.04% v/v) at 37 °C for ∼4 h. The *A*_650_ was measured, and the cells were normalized to 2 × 10^9^ CFU/ml before infection. Infection was performed as previously described (39). Briefly, the normalized cells were used to infect the Henle-407 cells and centrifuged at 105*g* for 10 min. After centrifugation, the 96-well plate was incubated at 37 °C with 5% CO_2_ for 30 min. After 30 min, the nonadherent population was removed by washing four times with PBS. After washing, 100 μl fresh tissue culture media (FluoroBrite Dulbecco's Modified Eagle's Medium; Thermo Fisher Scientific A1896701) with Kan, Amp, Gen, IPTG, 10 mM Hepes, and 1:1000 dilution Hoechst was added to each well and was immediately imaged for 10 h with half an hour interval. Samples were incubated at 37 °C during imaging. Our positive control VCA0956 was used to setup the exposure settings for Hoechst (blue channel), the tetramethylrhodamine (red channel), and the phase contrast. Settings for analysis and calculations were same as detailed above.

### Acid resistance (AR1 and AR2)

AR was setup as previously described with modifications (41, 44). *S. flexneri* strains were grown overnight in LB media with Kan at 30 °C for ∼16 h. Next day, for AR1 system the strains were subcultured 1:100 in LB media with 50 mM MES, Kan, and IPTG at pH 5.5 overnight at 37 °C with shaking. For AR2, strains were subcultured in LB media with 50 mM MES, 0.4% glucose, Kan, and IPTG at pH 5.5 overnight at 37 °C with no shaking. The following day, the *A*_650_ was measured for all the strains (AR1 and AR2) and the bacterial cells were normalized to 5 × 10^8^ CFU/ml. The normalized cells were acid shocked in M9 media at pH 2.5 at 37 °C (AR1) and M9 media at pH 2.5 with 50 mM glutamate at 37 °C (AR2). As a control, each strain was also incubated in M9 media at pH 7. After 1 h, cells were plated with serial dilution. Colony counts and the dilution factor was recorded to calculate the percent survival, determined by dividing the CFU/ml of acid shocked strains by the CFU/ml of the same strains incubated in pH seven medium.

### Cell culture assays

The invasion assay was set up as previously described (39). Briefly, 1 day before experiment, Henle-407 were split in 6-well plates for ∼10% confluency. *S. flexneri* DGC strains were inoculated in LB media with Kan and incubated overnight at 30 °C. Each strain was subcultured 1:100 in LB media with Kan, IPTG, and 0.04% DOC for ∼4 h. The *A*_600_ was normalized to 2 × 10^9^ CFU/ml and 100 μl of suspension was added to individual wells to infect Henle cells. Plates were centrifuged for 10 min at 1000 rpm and then incubated for 30 min at 37 °C with 5% CO_2_. Each well was washed four times with PBS and incubated again with Gentamycin for 45 min. After washing the cells twice with PBS, each well was stained with Giemsa stain for 5 min and washed with water to remove excess stain. Later, 300 cells were counted for positively invaded cells with three or more bacterial cells.

Plaque assay was setup using previously described protocols (39). Briefly, 2 days before experiment, Henle-407 cells were split in 6-wells plates for ∼80% confluency. Overnight grown strains were subcultured 1:100 in LB with Kan for ∼4 h. The cells were normalized to 5 × 10^4^ CFU/ml, and 100 μl was added to individual wells. Each 6-well plate was centrifuged and incubated for 45 min at 37 °C with 5% CO_2_. Each well was washed four times with PBS and incubated with Kan, IPTG, and 20% glucose for 48 h. After incubation, wells were washed twice with PBS and stained with crystal violet for 2 min. All plates were later imaged and plaque size was measured using ImageJ software (https://imagej.net/ij/).

### Immunoblotting

Immunoblots were performed as previously described (20). Bacterial cultures were grown in LB broth at 37 °C. When the cells reached the mid log phase of growth, the *A*_600_ was read, and cells were collected by centrifugation and resuspended in SDS sample buffer (5% β-mercaptoethanol, 3% (wt/vol) SDS, 10% glycerol, 0.02% bromophenol blue, 63 mM Tris-Cl, pH 6.8) at a concentration of 2 × 10^9^ CFU/ml or 4 × 10^9^ CFU/ml (for *dgcN* and *dgcE* only). Samples were boiled for 10 min and then electrophoresed in a 20% SDS-PAGE gel. After electrophoresis, proteins were transferred to a 0.45-μm pore size nitrocellulose membrane (Hybond-ECL; GE Healthcare) and incubated with a α-HIS antibody (Genscript A00186), followed by horseradish peroxidase-conjugated α-mouse antibody (Abclonal AS003). Signals were detected by developing the blot with a Pierce ECL detection kit (Thermo Fisher Scientific). Protein domain predictions were made using MistDB (76).

## Data availability

We confirm that the data supporting the findings of this study are available within the article and its supplementary materials, and raw data are available from the corresponding author (B. K.) upon request.

## Supporting information

This article contains supporting information (38).

## Conflict of interest

The authors declare that they have no conflicts of interest with the contents of this article.

## Figures and Tables

**Figure S7:**