# Supplementary material for: Intact and mutated Shigella diguanylate cyclases increase c-di-GMP
Source: J Biol Chem. 2024 Jul 1;300(8):107525. doi: 10.1016/j.jbc.2024.107525 (PMC11327459; doi:10.1016/j.jbc.2024.107525)
Supplement: Supporting Files [file mmc1.docx]

**Supporting Data - Tables**

| **Table S1 - Strains and plasmids used for this study.** | | | | | | | |
| --- | --- | --- | --- | --- | --- | --- | --- |
| **Strain** | **Parent Strain** | | **Other information** | | **Method for chromosomal mutation** | **Reference** | |
| *Shigella flexneri* 2457T |  | |  | |  |  | |
| Δ4DGC *S. flexneri* | *S. flexneri* 2457T | | Deletion of *dgcC, dgcF, dgcI,* and *dgcP* | | Homologous Recombination |  | |
| Δ5DGC *S. flexneri* | Δ4DGC *S. flexneri* | | Deletion of *dgcC, dgcF, dgcI, dgcP,* and *dgcE* | | Homologous Recombination |  | |
| Δ6DGC *S. flexneri* | Δ5DGC *S. flexneri* | | Deletion of *dgcC, dgcF, dgcI, dgcP, dgcE*, and *dgcQ* | | Homologous Recombination |  | |
| *E. coli* BL21 |  | |  | |  |  | |
| **Plasmids** | | | | | | | |
| **Plasmid** | | **Parent Plasmid** | **Other information** | **Antibiotic marker plasmid** | | | **Reference** |
| *dgcC* expression | | pEVS143 | pTac promotor (IPTG inducible) + Sf *dgcC* | Kanamycin | | |  |
| *dgcF* expression | | pEVS143 | pTac promotor (IPTG inducible) + Sf *dgcF* | Kanamycin | | |  |
| *dgcI* expression | | pEVS143 | pTac promotor (IPTG inducible) + Sf *dgcI* | Kanamycin | | |  |
| *dgcP* expression | | pEVS143 | pTac promotor (IPTG inducible) + Sf *dgcP* | Kanamycin | | |  |
| GG→AA *dgcC* | | pEVS143 | pTac promotor (IPTG inducible) + Sf *dgcC* GG→AA | Kanamycin | | |  |
| GG→AA *dgcF* | | pEVS143 | pTac promotor (IPTG inducible) + Sf *dgcF*→GGAA | Kanamycin | | |  |
| GG→AA *dgcI* | | pEVS143 | pTac promotor (IPTG inducible) + Sf *dgcI* GG→AA | Kanamycin | | |  |
| GG→AA *dgcP* | | pEVS143 | pTac promotor (IPTG inducible) + Sf *dgcP* GG→AA | Kanamycin | | |  |
| Riboswitch Reporter | | RSF1010 |  | Ampicillin | | | (38) |
| *dgcM* expression | | pET 28a(+) | T7 Promotor (IPTG inducible) | Kanamycin | | |  |
| *dgcN* expression | | pET 28a(+) | T7 Promotor (IPTG inducible) | Kanamycin | | |  |
| *dgcE* expression | | pET 28a(+) | T7 Promotor (IPTG inducible) | Kanamycin | | |  |
| *dgcQ* expression | | pET 28a(+) | T7 Promotor (IPTG inducible) | Kanamycin | | |  |
| *dgcO* expression | | pET 28a(+) | T7 Promotor (IPTG inducible) | Kanamycin | | |  |
| *dgcJ* expression | | pET 28a(+) | T7 Promotor (IPTG inducible) | Kanamycin | | |  |
| *pdeR* expression | | pET 28a(+) | T7 Promotor (IPTG inducible) | Kanamycin | | |  |

| **Table S2 - Primers used in this study** | | |
| --- | --- | --- |
| **Target Gene** | **Primer sequence** | **Strain constructed** |
| *dgcC*_KO | Fw-ATAGAATTCAGGAGCTAAGGAAGCTAAAATGTTCCCAAAAATAATGAATGAT  Rv- ATAGGATCCTCAGGCCGCCACTTCGG | *dgcC* expression strain on pEVS143 |
| *dgcF*_KO | Fw-ATAGAATTCCAGGAGCTAAGGAAGCTAAATTGCTTGCGGACTGGTTCAG  Rv-ATAGGATCC TCAGACAACTTCCTCACCGTAAC | *dgcF* expression strain on pEVS143 |
| *dgcI*_KO | Fw- ATAGAATTCCAGGAGCTAAGGAAGCTAAAATGTCCAGAATCAATAAGTTCGTAC  Rv-ATAGGATCCTTACTGCCAGCTAATCTGTGC | *dgcI* expression strain on pEVS143 |
| *dgcP*_KO | Fw- ATAGAATTCAGGAGCTAAGGAAGCTAAAGTGTCAGATCAGATTATCGCCC  Rv-ATAGGATCCTCAGGAATGTAGCGCTGGATG | *dgcP* expression strain on pEVS143 |
| GG→AA *dgcC* | Fw- TTGGTCGGTTTGCCGCCGATGAGTTTGCAGTAATCA  Rv- GCAAACTCATCGGCGGCAAACCGACCAATCACATCG | *dgcC* GG→AA strain on pEVS143 |
| GG→AA *dgcF* | Fw- GCATGGCCGCCGAAGAATTTGCTGTTGCAGTGCCG  Rv- CTTCGGCGGCCATGCGCGCCACCAGCCC | *dgcF* expression strain on pEVS143 |
| GG→AA *dgcI* | Fw- TCTACCGCTTCGCAGCTGACGAATTTGCGGTGATTT  Rv- GCAAATTCGTCAGCTGCGAAGCGGTAGACTTTACCT | *dgcI* expression strain on pEVS143 |
| GG→AA *dgcP* | Fw- GAAGTTATTGGTCGTCTCGCTGCTGATGAGTTTTTGGTTGTT  Rv- ACCAAAAACTCATCAGCAGCGAGACGACCAATAACTTCGCCA | *dgcP* expression strain on pEVS143 |
| pEVS143 seq | Fw- CGGTTCTGGCAAATATTCTG  Rv- TAATTGGGGACCCTAGAGGT |  |
| ΔE DGC | Fw- CCCCATTATAAGGGGTACGAATAAAGTAACGGGGGTCCATATGAATATCCTCCTTAGTTC  Rv- GGCAATGGGCAAACGCTCTCTCAGTTAATCGCGAAATAACTGTAGGCTGGAGCTGCTTCG | Δ5 DGC |
| ΔQ DGC | Fw- TGCCAGAATCATAAAAAAGCAGGTTGGGAGTCGTGTCCATATGAATATCCTCCTTAGTTC  Rv- GACCATTTTTTCTCCGCCCGTTAAGCGTTATCGCTCGCGATGTAGGCTGGAGCTGCTTCG | Δ6DGC |
| *dgcC* seq | Fw- CCTTTAGCCCCGTCTCTATA  Rv- ATCAGAAAAACTCAGCAAATCCT |  |
| *dgcF* seq | Fw- GAACATGCTATCCTTTATGAGAATTT  Rv- CGAGGAAATACGCCTTTTCCTGGATC |  |
| *dgcI* seq | Fw- GGGGTATCTGTACCAGAAACCCATG  Rv- GAGTACGATTTGTGGGGTAGCCG |  |
| *dgcP* seq | Fw- CCGCTGTAATGAAGATTACAACCG  Rv- CGTCTTGTTTCATCTTTGTTGATG |  |
| *dgcE* seq | Fw- CACCAGACCCGGTGACATCA  Rv- GCCTTATCCTGCCTGCGGAA |  |
| *dgcQ* seq | Fw- ATAGTCGCGTCTTATCATGCC  Rv- CCGGCCCGCGTCTGGCGAAC |  |
